# Importance of Skeletal Muscle and Interdisciplinary Team Approach in Managing Pneumonia in Older People

**DOI:** 10.3390/jcm12155093

**Published:** 2023-08-03

**Authors:** Satoru Ebihara, Tatsuma Okazaki, Keisuke Obata, Takae Ebihara

**Affiliations:** 1 Department of Internal Medicine and Rehabilitation Science, Tohoku University Graduate School of Medicine, Sendai 980-8574, Japan; tmokazaki0808@gmail.com (T.O.); keisuke.obata1110@gmail.com (K.O.); 2 Department of Geriatric Medicine, Graduate School of Medicine, Kyorin University, Tokyo 181-8611, Japan; takae-ebi@ks.kyorin-u.ac.jp

Pneumonia is the most frequent lower respiratory tract disease and a major cause of morbidity and mortality globally [1]. Several attempts have been made to categorize it based on origin, clinical setting in which the patient contracts it, and pattern of lung parenchyma involvement, and others. The classification of pneumonia is becoming more complex with the increasing diversity of patient populations.

The most common classification of pneumonia is based on the location where pneumonia occur. Community-acquired pneumonia occur at the community and hospital-acquired pneumonia occur at the hospital. Pneumonia among residents of nursing homes or long-term care facilities is categorized as nursing-home-acquired pneumonia. This concept is based on the idea that the causative bacterium of pneumonia is determined to some extent by the location where pneumonia occur and serves as a guideline for the use of antibiotics [2,3,4]. Guidelines have been established for each type of pneumonia. However, deaths from pneumonia have continued to increase annually with the population aging, even after the publication of these guidelines [1]. This indicates that the guidelines for pneumonia treatment should not only be focused on the use of antibiotics.

Aspiration pneumonia is the most dominant form of pneumonia in older adults [5]. Many studies have been conducted on dysphagia, aspiration, and countermeasures. However, not all cases of aspiration result in pneumonia, and the development of pneumonia involves several steps, such as coughing, spitting, cilia in the airways, handling foreign bodies in the airways, and immunity [6]. Few studies have discussed countermeasures linking aspiration to pneumonia. In this special issue, six original studies and four review articles can be found that provide new insights into pneumonia in older people. 

Serial studies concerning the relationship between forced vital capacity (FVC) on spirometry and death due to pneumonia in older people have been published by the same laboratory in Japan. In the first study, the authors analyzed the data of a large cohort and found that low FVC was associated with a high all-cause mortality rate of community-dwelling older people [7]. In the second study, the authors reported a stronger relationship between FVC and pneumonia mortality than between FVC and all-cause mortality [8]. They suggested the involvement of impaired leg muscle strength in pneumonia-related deaths. In addition, researchers in Denmark reported that physical inactivity and sedation during and after admission of older patients with aspiration pneumonia were related to mortality [9]. Ichibayashi et al. showed that the maximum tongue pressure after extubation can predict post-extubation aspiration pneumonia in older patients receiving mechanical ventilation [10]. 

Taken together, the above four reports indicate a strong relationship between impairment of skeletal muscle and pneumonia or pneumonia-related death of older adults. Mendes and colleagues showed that healthcare-associated pneumonia is the main causes of hospital admission and death among patients with pneumonia, the majority of which were empirically treated, in Portugal [11]. The high frailty ratio of patients with healthcare-associated pneumonia in this study also suggested a strong relationship between a decline in skeletal muscle function and pneumonia-related death. A systematic review showed that pneumonia is a clinically relevant complication in patients with spinal cord injury and prevention should be targeted at patients with tetraplegia, suggesting the importance of skeletal muscles in the development of fatal pneumonia [12]. 

The remaining four papers in this issue highlight the importance of comprehensive functional assessment and interdisciplinary team care, which is beyond the use of antibiotics, in older patients with aspiration pneumonia. Yoshimatsu et al. reported no evidence of the benefit of anaerobic coverage in the antibiotic treatment of aspiration pneumonia [13]. They also reported that the simplistic and knee-jerk diagnosis of aspiration pneumonia is not based on important investigations and functional assessments, resulting in inappropriate patient management [14]. These reports suggest that careful assessment plays a pivotal role in the management of aspiration pneumonia. Okuni and Ebihara showed that dysphagia screening tests, such as the water swallowing test, were useful in predicting future aspiration, and an interdisciplinary team approach may be effective in preventing aspiration pneumonia [15]. However, they could not determine what kind of team approach was most effective in preventing aspiration pneumonia in older adults. A review by Ebihara provides some hints. The review stated that the team approach to managing aspiration pneumonia differed depending on the time axis of disease progression in managing aspiration pneumonia [16]. 

Since this special issue reveals the importance of skeletal muscle and an interdisciplinary team approach, it will help clinicians make decisions and treatment choices. We appreciate the valuable contributions of all authors. We are also grateful to the reviewers for their professional and constructive comments and to the JCM team for their continuous support with this special issue.
